# The Attitude of Great Writers Towards Disease

**Published:** 1893-02-04

**Authors:** 


### The Attitude of Great Writers Towards Disease.

### XX.—THE PLAGUE AT MILAN, 1630.

It is curious to find some of the most accurate accounts we possess of plague visitations inserted among love tales. Manzoni was but following in Boccaccio's steps when he framed his account of the plague at Milan in the story of the "Betrothed Lovers." He was not, like Boccaccio, an eye-witness, but his account was gathered from contemporary documents never before critically examined and compared, and gives a remarkable picture of a state of peculiar panic never surpassed among stories of similar seasons of alarm.

At Milan this panic began under the dangerous form of entirely refusing to believe in the plague at all until it was raging in their midst. The usual warnings were not wanting. In October, 1629, the first cases occurred in the environs of the city, and were duly reported. In November the first death took place within the walls, and throughout the winter isolated cases showed that the infection was smouldering. Yet neither the governing body nor the citizens would hear the mention of the truth. With incredible blindness they treated the doctors, who laboured incessantly to awake them to a sense of their danger, as malicious disseminators of false reports, so that more than once the lives of these devoted men were in peril from the populace. By the end of March the deaths had become frequent, and when the rich, too, began to be attacked, there seemed no longer room for doubt. Dr. Krocker, in the current number of the *Deutsche Rundschau*, quotes with particular emphasis Manzoni's comment on the general incredulity. "At first, then, not pestilence, absolutely no; on no account whatever; forbidden even to mention the word. Then pestilential fever; the idea is regarded askance by means of an adjective. Then not true pestilence; that is to say, certainly pestilence, but only in a kind of way; not exactly pestilence, but a thing for which it is difficult to find another name. Finally, pestilence without doubt and without debate." The authorities had been convinced long before the populace, and during the Whitsuntide festivities they hit upon the horrible expedient of parading through the streets, crowded with holiday makers, the exposed bodies of a whole family who had died that morning of plague.

In order to justify previous scepticism, an idea was now started that the plague had been deliberately introduced from Madrid by the agency of four Frenchmen. A despatch was freely quoted in which Philip IV. warned the governor of Milan against these Frenchmen, who had escaped from Madrid under suspicion of being "untori," or smearers of plague ointment. One evening in May late watchers in the cathedral declared they had seen persons smearing with ointment a screen or barrier which served to divide the sexes. The President of the Council of Health promptly ordered the neighbouring seats to be cleansed, while it was confidently believed in the city that every part of the cathedral, even to the bell-ropes, had been infected. The following morning the terror-stricken citizens awoke to find the doors and walls in every part of the town smeared in long lines with a ghastly-looking yellowish white composition. Whether it were a ribald trick, or a more sinister attempt to increase public confusion, can never be known. The fact is attested beyond all possibility of doubt. The city was beside itself with terror. Householders attempted with burning straw to rid their houses of the ominous markings. People shuddered and crossed themselves as they met in the streets, while unhappy foreigners who ventured to show their faces were seized by the excited mob to be haled off to prison—lucky if they reached it alive. The wildest stories were circulated as to the composition of this plague ointment. It was said to be made up of toads, serpents, foam from the lips of plague patients, and every horror which the most diseased imagination could suggest. It is easy to understand the harm done by such theories. Every sanitary precaution was scorned, the doctors' warnings were persistently disregarded, and public attention was concentrated solely on the means of discovering the "smearers." In the height of the panic no one was safe from the popular fury. In the church of San Antonio a poor old man who was observed to dust one of the seats with his cloak before sitting down, was set upon by the infuriated congregation with cries of "He is smearing the benches," and was dragged outside to be stoned and beaten to death in a few moments.

In the hope of turning aside the excitement, the Archbishop consented at length to a solemn procession through the city of the sacred relics of San Carlo, in which all might join. Some five hundred infected houses were closed, and the occupants forbidden to take part in the solemnities. On June 11th the procession, consisting of nearly all the inhabitants, passed through all quarters of the city to the solemn service of intercession at the cathedral. Alas, the following day the deaths in every part of the city increased with a bound, for which the procession alone could be held responsible. But the popular frenzy had an answer for all who urged the madness of such assemblies in times of contagion. "The streets had been sprinkled over night with plague powder, and the smearers had been seen busy in the procession infecting all whom they could approach."

From this date the disease spread with terrible rapidity. By the beginning of July the daily mortality was 500, from which it went on increasing to 1,200, 1,500, and even, according to one authority, 3,500. Something must be allowed for exaggeration at a time when no official registration was possible.

Meantime, what care was it found possible to bestow on the sick? Almost from the first the lazaretto had been placed under the care of the Capuchin monks, headed by Father Felice. This devoted man speedily succeeded in reducing to order the hospital given up before his appearance to panic and licence. He soon took the plague himself, but recovered, and lived to carry through to the end the noble work to which he had set his hand. Most of his assistants died at their post, but to the honour of the Capuchin order, be it said, there were never wanting fresh recruits to fill the vacant places. Some idea of the difficulties under which they laboured may be gathered from the fact that the number of sick persons in the lazaretto rose to 12,000. The accommodation was indefinitely extended by a series of rude tents, for though supplementary hospitals were actually planned and begun, the workmen to finish them were unobtainable. Very often the supply of food and of the commonest necessaries failed altogether, and famine stared them in the face, when some unexpected donation would come to relieve the worst necessities. Once the hospital was left entirely without doctors, until some were bribed by large payments to give their services.

In the City the usual scenes noted at such times were observable. Hundreds of babies, whose mothers were plague-stricken, died of exposure and desertion. Every function of civil government was thrown into the wildest disorder. The authorities were pitiably powerless to cope with the evil, and the worst excesses were committed by the officials, gathered from among the lowest of the people, who were appointed to regulate the burials, and the isolation of the sick.

But the leading feature of this plague which marks it out from all others was the "madness, the monstrosity of the suspicions. Not only against neighbours, against friends, and guests, was suspicion roused, but those ties of human love, husband and wife, father and son, brother and sister, were names of terror, while the domestic hearth and common table were shunned for the hidden poison lurking in their midst." The delirious confessions of many dying patients were held to confirm the "smearing" theory, which gained credence even with the gravest authorities. And because the malice of man was not held sufficient for the ravages of the plague, it began to be attributed to diabolical agency. One dying man related that several persons had visited him by night, offering him money and health if he would smear the adjoining houses. On his refusal they disappeared, leaving in their stead till dawn three hideous cats on the bed and a wolf beneath it. Such stories, with many monstrous variations, were freely circulated, and almost universally believed. And as though death were not sufficiently glutted with his feast, the torture and execution of persons convicted of disseminating the plague became common.

It would seem, then, that even overwhelming apprehension of advancing danger is less to be deprecated than determined disregard of it. The nonchalance which ignores, is only too likely to pass away into the panic which feeds, the pestilence. But the worst that can be said for even excessive alarm about the future is that it gives rise to measures of precaution, which may prove useless, but can hardly under any circumstances be pernicious.

